# 46213 Florida Community-Engaged Research Alliance Against COVID-19 in Disproportionately Affected Communities (FL-CEAL): addressing education, awareness, access, and inclusion of underserved communities in COVID-19 research

**DOI:** 10.1017/cts.2021.609

**Published:** 2021-03-30

**Authors:** Olveen Carrasquillo, Erin Kobetz-Kerman, Victoria Behar-Zusman, Sheela Dominguez, Mario DeLa Rosa, Elena Bastida, Eric Wagner, Adrianna Campa, Folakemi Odedina, Elizabeth Shenkman, Cynthia Harris, Katherine Chung-Bridges, Timothy Long

**Affiliations:** 1 University of Miami; 2 Florida International University; 3 University of Florida; 4 Florida A&M University; 5 Health Choice Network

## Abstract

ABSTRACT IMPACT: Understanding the needs and barriers or facilitators to participation in research, especially among minority communities is critical not only for COVID-19 research but also for future clinical and translational research and health disparities studies. OBJECTIVES/GOALS: The overall goal of this project is to enhance education, awareness, access, and inclusion of underserved communities across Florida in COVID-19 research, especially among Black and Hispanic minority groups that are disproportionately affected by COVID-19. METHODS/STUDY POPULATION: Through strategic partnership among five academic institutions and community-based organizations across the state of Florida, the FL-CEAL team will implement focus groups and surveys in minority communities in Florida to gauge the awareness and understanding of COVID-19, and the barriers and facilitators for participation in COVID-19 research studies. These communities include but are not limited to Latinx and Black populations in South and Central Florida, and Black communities in North Florida. The outcomes will help shape strategies for outreach and dissemination activities and minority recruitment plans to promote participation of minorities into vaccine and therapeutic trials. RESULTS/ANTICIPATED RESULTS: An estimated 75-125 participants will be recruited for focus groups. Four focus groups with minority communities have been conducted and the results are being analyzed. A common Community-Based Needs Assessment survey is being finalized and will be deployed across the 11 states that are part of the national CEAL consortium. Community Health Workers are being engaged to support outreach and dissemination to educate targeted communities on COVID-19 research and the importance of participation in COVID trials. To date, 243 CHWs and 880 community members have been engaged. Minority participation in COVID-19 vaccine trials at University of Miami has been higher than the national average. DISCUSSION/SIGNIFICANCE OF FINDINGS: The FL-CEAL Alliance has successfully demonstrated a coordinated effort to engage minority communities affected by COVID. Through strategic geographic partnerships, FL-CEAL will positively impact minority communities throughout the state that has one of the most diverse populations in the nation.

